# Case-fatality and hospitalization rates for dermatological diseases in Brazil in the context of the COVID-19 pandemic^☆^^☆☆^

**DOI:** 10.1016/j.abd.2020.12.002

**Published:** 2021-03-22

**Authors:** Vanessa Barreto Rocha, Claudia Cristina de Aguiar Pereira, Leticia Arsie Contin, Carla Jorge Machado

**Affiliations:** a Hospital das Clínicas, Belo Horizonte, MG, Brazil; b Fundação Oswaldo Cruz, Escola Nacional de Saúde Pública Sergio Arouca, Rio de Janeiro, RJ, Brazil; c Hospital do Servidor Público Municipal de São Paulo, Clínica de Dermatologia, São Paulo, SP, Brazil; d Departamento de Saúde Pública, Faculdade de Medicina, Universidade Federal de Minas Gerais, Belo Horizonte, MG, Brazil

Dear Editor,

Dermatology is mainly an outpatient specialty. Hospital admissions are uncommon; however, they can have important impacts on morbidity and mortality. There are few studies on the rates of hospitalization and mortality from dermatological diseases, mainly in Brazil.^1^, ^2^

Amidst the COVID-19 pandemic, several medical specialties reported that patients have been postponing their routine and follow-up appointments. This behavior has led patients to seek care only when they achieve a severe condition.^3^, ^4^, ^5^, ^6^

To date, there have been no published reports on how the pandemic has changed this scenario in dermatology in Brazil. This ecological study aims to investigate hospitalizations and deaths from dermatological diseases during the period of the COVID-19 pandemic, comparing the case-fatality findings with those related to the five-year period before the pandemic. The hypothesis is that, in Brazil, case-fatality rates from dermatological diseases increased in 2020, when compared to 2015–2019. Official in-hospital morbidity data are available from DATASUS (Information Technology Department of the Unified Health System - www.datasus.saude.gov.br), through the SIH (Hospital Information System), which describes hospitalizations of all public hospitals and represents more than 75% of total hospitalizations (public and private) in Brazil.

Table 1 summarizes hospitalizations and deaths due to dermatological diseases between March and August (6 months) from 2015 to 2019 and of the same months in 2020. From these data, it was possible to obtain in-hospital case-fatality rates, as well as case-fatality relative risks in 2020 (as compared to 2019).

**Table 1:** Hospitalization and mortality rates for dermatological diseases in Brazil in March and April (2015 to 2019) when compared to the same months of 2020, in the context of the COVID-19 pandemic.

Codes and descriptions ICD-10	Hospitalizations from March to August (A)		Deaths from March to August (B)		Case fatality (B)×100/(A)		Relative Risk (95%CI)	p-value for the relative risk
	2015 to 2019 (mean)	2020	2015 to 2019 (mean)	2020	2015 to 2019 (mean)	2020		
C43-C44; D22-D23 Skin Neoplasias	28,865	21,926	485	508	1.68	2.32	1.38 (1.22;1.56)	<0.001
--C43-C44 Melanoma and Other Malignant Skin Neoplasias	24,293	19,929	484	506	1.99	2.54	1.27 (1.13;1.44)	<0.001
--D22-D23 Benign skin neoplasias	4,572	1,997	1	2	0.02	0.10	4.58 (0.42;50.5)	0.172
L00-L99 Diseases of the skin and subcutaneous tissue	131,325	90,182	2.004	1.947	1.53	2.16	1.42 (1.33;1.51)	<0.001
--L00-L08 Infections of the skin and subcutaneous tissue	46,725	35,034	506	514	1.08	1.47	1.36 (1.19;1.53)	<0.001
--L00-L09 Other diseases of the skin and subcutaneous tissue	84,601	55,148	1.498	1.433	1.77	2.60	1.47 (1.37;1.58)	<0.001
Total	160,191	112,108	2.489	2.455	1.55	2.19	1.41 (1.33;1.49)	<0.001

Note: The p-values for relative risks associated with case-fatality rates were obtained based on the Chi-Square distribution (Stata / SE software, version 12.0 for Mac, csi command).

There was an increase in all dermatological case-fatality rates in relation to the average of March and April in the years 2015 to 2019, except for benign skin neoplasia (p = 0.214). Except for this disease, the hypothesis of the present study was confirmed for all others. Moreover, the relative risks of dermatological in-hospital case-fatality ranged from 1.27 to 1.47 (p < 0.001), indicating case-fatality rates ranging from 27% to 47%, which is higher than in the first 6 months of 2020, when compared to the same 6 months in 2020.

As study limitations, there is a lack of stratification of dermatological diseases in the data, due to the way the diseases are tabulated in DATASUS, with many generic ICDs. There is also the possibility of problems with ICD registration and a large number of undetermined cases among the registries.

Dermatology includes diseases with high morbidity and mortality, such as pemphigus, drug reactions, autoimmune diseases, some types of infection, and cancer. They cannot be neglected, even during the emergence of COVID-19. Only the systematic individualized study of deaths from dermatological diseases during the COVID-19 pandemic can corroborate the hypothesis raised in the present study. However, the results reflect the importance of specialized, in-hospital assistance in the care of severe dermatoses. Thus, it is important that patients with severe conditions should be followed, even if it is at a distance.

As in other specialties, a side effect of the public health crisis caused by COVID-19 is the delay in disease diagnoses, often due to fear of contracting SARS-CoV-2 in healthcare settings. The postponing of demand for care can worsen the general health status of patients, leading to death. In Brazil, an additional factor that can promote this behavior is the unprecedented strain on the health system, due to the demands caused by the large numbers of COVID-19 patients.^7^

## Financial support

None declared.

## Authors’ contributions

Vanessa Barreto Rocha: Conception and design of the study; collection, analysis and interpretation of data; writing and critical review of the manuscript; approval of the final version of the manuscript.

Claudia Cristina de Aguiar Pereira: Conception and design of the study; collection, analysis and interpretation of data; critical review of the manuscript; approval of the final version of the manuscript.

Leticia Arsie Contin: Conception and design of the study; collection, analysis and interpretation of data; critical review of the manuscript; approval of the final version of the manuscript.

Carla Jorge Machado: Conception and design of the study; data collection, analysis and interpretation; critical review of the manuscript; approval of the final version of the manuscript.

## Conflicts of interest

None declared.
